# Smartphone Distraction and Academic Anxiety: The Mediating Role of Academic Procrastination and the Moderating Role of Time Management Disposition

**DOI:** 10.3390/bs14090820

**Published:** 2024-09-14

**Authors:** Yuanting Jin, Wanqi Zhou, Yueling Zhang, Zeyang Yang, Zaheer Hussain

**Affiliations:** 1 Department of Psychology, School of Education, Soochow University, Suzhou 215123, China; 2 School of Social Sciences, Nottingham Trent University, Nottingham NG1 4BU, UK

**Keywords:** smartphone distraction, academic anxiety, academic procrastination, time management disposition, moderated mediation

## Abstract

The present study investigated the relationship between smartphone distraction, academic procrastination, academic anxiety, and time management disposition. A total of 474 college students were recruited to complete a survey comprising measures of smartphone distraction, academic procrastination, academic anxiety, and time management disposition. The hypothesised moderated mediation model was tested using Model 4 and Model 15 of the PROCESS macro for SPSS. Results showed that smartphone distraction was positively and significantly correlated with academic anxiety (r = 0.40, *p* < 0.001) and academic procrastination (r = 0.42, *p* < 0.001). Academic procrastination mediated the relationship between smartphone distraction and academic anxiety. Time management disposition moderated the paths from academic procrastination and smartphone distraction to academic anxiety. The present study suggests that smartphone distraction could predict increased levels of academic procrastination, which could then lead to higher academic anxiety. However, the predicting effects in this mediation model could fluctuate across individuals with different time management dispositions. Further studies are needed to explore the mechanism of smartphone distraction using different methods.

## 1. Introduction

The number of global smartphone subscriptions reached 6.6 billion in 2022 [1]; these powerful computing devices are now prominent in society which has led to increased interest among researchers. Many recent studies have assessed the relationship between problematic smartphone use (PSU) and its potential consequences such as anxiety, depression and procrastination [2,3,4,5,6,7,8]. However, as a key component of PSU [9], smartphone distraction was scarcely investigated in previous smartphone studies. Smartphone distraction can be described as attention discontinuity caused by internal (e.g., worries, fear of missing out) triggers or external (e.g., smartphone notifications) signals or the conflict of the two [9]. Studies have shown the link between smartphone distraction, PSU, and mental health risk factors such as anxiety [8,10], and the link between PSU and procrastination [3,11,12,13]. However, little is known about how smartphone distraction could affect procrastination and anxiety in academic situations. Moreover, studies also show that self-regulation skills such as time management and self-control could moderate the relationship between technology use, PSU, and mental health and well-being [14,15]. Therefore, it is possible that individuals with different levels of self-regulation skills (e.g., time management) could be influenced differently by smartphone distraction. Thus, the present study aims to explore the relationship between smartphone distraction, academic procrastination, academic anxiety, and time management.

## 2. Literature Review

### 2.1. Smartphone Distraction and Academic Anxiety

Research has reported associations between smartphone distraction, problematic smartphone use (PSU) [9,10], and negative psychological well-being [16]. PSU can be defined as the uncontrolled use of smartphones with consistent cravings that result in functional impairment among users [4,17]. Terms such as addiction, dependence, and problematic use have been used to describe the uncontrolled use of smartphones, which leads to functional impairment [17]. However, since not all smartphone use behaviours could reach the level of addiction, the present study uses the term PSU to describe maladaptive smartphone usage. The theoretical pathway model suggests that PSU has three types—addictive use, antisocial use, and risky use—and distraction appears to be the core component since individuals could feel compelled to use smartphones in prohibited situations (e.g., driving) due to fear of missing out, low self-control, or impulsivity [17]. In a qualitative study involving 265 British undergraduates, distraction was reported as one of the major negative effects of PSU, along with other related factors, such as procrastination, time-wasting, and disrupted social relationships [18]. It appears that smartphone distraction is a key component of PSU that needs more research attention.

Empirical studies largely prove that PSU is associated with mental health factors such as anxiety and depression [2,4,8,19]. Qualitative evidence also shows that PSU can result in decreased concentration, lower productivity, reduced efficiency, and sleep disturbances [18]. Individuals might struggle to cope with these negative impacts, leading to increased anxiety [17]. Research evidence on smartphone distraction and mental health is still scarce. Some studies have found that smartphone distraction can predict mental health issues such as anxiety [10,16,20]. For example, Chu et al. found that smartphone distraction predicted lower psychological well-being among 914 university students [16]. Similarly, also among college students, another study revealed that smartphone distraction was positively correlated with anxiety and predicted anxiety in a path model through PSU as a full mediator [10]. The study concluded that high levels of smartphone distraction can indicate high levels of PSU, which could predict anxiety disorders. Based on the empirical studies, it is possible that problematic smartphone users can easily be distracted by their devices, leading to negative consequences in daily life, such as interrupted study sessions [13]. The negative consequences may increase their level of anxiety.

The trait-state anxiety theory proposes that anxiety can be divided into trait anxiety (dispositional) and state anxiety (situational), and state anxiety is the result of an individual’s cognitive appraisal of internal stimuli, such as personal needs, or external stimuli, such as stressors [21]. The recent control-value theory of achievement emotions suggests that academic anxiety, one of the emotions experienced in achievement or academic situations, is derived from an individual’s appraisal of negative academic outcomes (e.g., failure in exams) and learning and social environments [22]. Students could feel anxious in academic situations such as class, learning, and during tests [22]. Therefore, situational-specific anxiety, such as academic anxiety, can be the result of complex internal and external factors. Given the potential link between smartphone distraction and general anxiety identified in previous studies [10,16,20], the effects of smartphone distraction in academic situations need to be further explored. Since previous studies about smartphone distraction mainly focused on students’ general anxiety, it is thus necessary to investigate the link between smartphone distraction and academic anxiety among students.

### 2.2. The Mediating Role of Academic Procrastination

The link between smartphone distraction and academic anxiety might be more complicated than previously thought since the antecedents of anxiety include a wide range of individual and environmental factors [22]. Given that the paths from smartphone distraction to mental health risks were found to be fully mediated through PSU [10], there might exist some psychosocial events or variables (besides PSU) that can bridge the link between smartphone distraction and academic anxiety. In other words, something could happen before academic anxiety and after smartphone distraction.

Academic procrastination has been defined as the intentional delay of tasks that need to be completed [23]. The theoretical model of academic procrastination suggests that the antecedents for academic procrastination include the task (e.g., task difficulty), the teacher (e.g., instruction skills), and the students (e.g., interest and organizational skills) [23]. Thus, if students have poor organizational skills or the task is too hard to concentrate on, the distractions of smartphones (e.g., notifications or being on the desk) may trigger academic procrastination. This is shown in many recent empirical studies where the relationship between PSU and procrastination was identified [3,5,6,7,11,12,13]. For example, PSU was found to positively predict academic procrastination among university students [7,12] and young children [13]. Some studies found that procrastination was a predictor of PSU [11]. A longitudinal study revealed a bi-directional but unstable relationship between PSU and procrastination [5]. Taken together, PSU appears to be an important risk factor for academic procrastination. Schraw et al. believed that academic procrastination can be predicted by individual factors such as self-organization skills [23]. Thus, it is possible that individuals with low self-organization skills or poor self-control may fail to finish tasks on time due to a craving to check their smartphones (e.g., social media) [7], leading to negative consequences, such as anxiety. However, there is a scarcity of research investigating smartphone distraction as a predictor of procrastination, though it has been noted as an important component of PSU [9,18].

Moreover, the theoretical model of academic procrastination [23] also suggests that academic procrastination could have a negative impact on life quality such as fatigue, stress, and anxiety. The link between procrastination and anxiety has been proved in many studies [24,25,26,27,28,29,30,31]. Some studies identified that anxiety was a predictor of procrastination [28,29]. For example, academic anxiety positively and significantly predicted academic procrastination in a structural equation model with good fit [29]. However, a longitudinal study among high school students found that academic procrastination positively and significantly predicted test anxiety two months later, but not vice versa [30]. Another longitudinal study reported that procrastination and anxiety were positively correlated at the beginning of the semester, but a decrease in academic anxiety during the semester was associated with an increase in procrastination [27], which shows the complexity and instability of this relationship. Therefore, it seems that academic procrastination and anxiety could predict each other in a bi-directional way but such a link still needs further investigation.

### 2.3. The Moderating Role of Time Management Disposition

Time management was conceptualized as a core component of self-regulation, which includes behaviours such as goal setting, time estimating, and effective use of time [32,33]. Time management disposition refers to a type of stable individual characteristic with three dimensions including time value, time monitoring, and time efficacy [34]. Time management disposition was reported to moderate the relationship between media multitasking and psychological well-being. For those with high levels of time management, media multitasking could negatively predict well-being, but for adolescents with low levels of time management, there was no significant predictive effect of media multitasking on well-being [14]. Time management disposition can also moderate the relationship between stress and anxiety of college students. For students with low levels of time management disposition, their personal and academic concerns could significantly predict anxiety, while the effect was not significant for students with higher levels of time management disposition [35]. Self-control, as another key component of self-regulation, apart from time management [32], was found to moderate the relationship between smartphone addiction and depression or anxiety [15]. For students with different levels of self-control abilities, the effect of PSU on mental health conditions can be different [15]. Therefore, for individuals with different levels of time management disposition or self-control abilities, the impact of the potential antecedents (e.g., media multitasking) on anxiety can be different.

Smartphone distraction, procrastination, and anxiety do not seem to be problems for individuals with better time management or self-regulation skills. Studies have shown that time management disposition was negatively associated with smartphone addiction and academic procrastination [36,37]. Similarly, students who are more confident in using self-regulation strategies reported lower levels of procrastination and anxiety [27]. Furthermore, time management skill was negatively associated with state and trait anxiety, and the improvement of time management skills can alleviate students’ state-trait anxiety [38]. However, to our knowledge, little study has investigated the relationship between smartphone distraction and time management. As noted above, smartphone distraction could be a key component of PSU [18]. Given the link between time management and smartphone addiction [36], it is possible that poor time management may result in greater distractions and, as a result, a higher level of academic anxiety. Since effective time management might reduce the need for procrastination [23], it can possibly change or moderate the pathways from smartphone distraction to procrastination and academic anxiety. Therefore, it is necessary to investigate the impact of time management disposition on the relationship between smartphone distraction, academic procrastination and academic anxiety.

### 2.4. The Present Study

**Figure 1:**
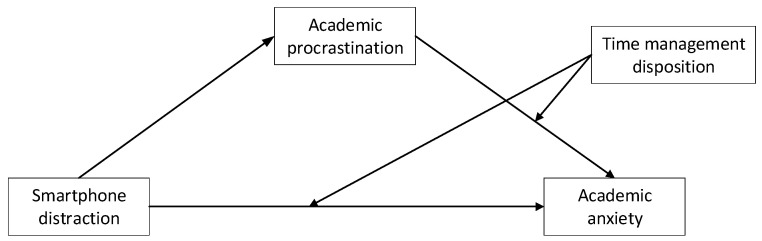
Hypothesised moderated mediation model.

According to the theoretical and empirical studies that have been reviewed [6,7,17,18,23,30,31], academic procrastination seems to be predicted by smartphone distraction and could predict academic anxiety. Namely, academic procrastination could mediate the relationship between smartphone distraction and academic anxiety. Moreover, for individuals with different time management disposition levels, the effects of smartphone distraction and academic procrastination on academic anxiety appear to be different based on existing evidence [14,35]. Therefore, the present study explores the relationship between smartphone distraction, academic procrastination, academic anxiety, and time management. Three hypotheses and a hypothesised moderated mediation model (see Figure 1) are proposed as follows.

**Hypothesis** **1** **(H1):** *Smartphone distraction will significantly and positively predict academic anxiety.*

**Hypothesis** **2** **(H2):** *Academic procrastination will play a mediating role in the relationship between smartphone distraction and academic anxiety.*

**Hypothesis** **3** **(H3):** *Time management disposition will play a moderating role in the direct path between smartphone distraction and academic anxiety, and in the second half of the mediating path (see H2).*

## 3. Materials and Methods

### 3.1. Participants

The present study recruited 474 Chinese university students using random sampling, convenience sampling and snowball sampling. There were 402 females (84.81%) and 72 males, ranging in age from 17 to 31 years (M = 21.25, SD = 2.04). The participants included 356 undergraduates (75%), comprising 23 first-year, 103 second-year, 127 third-year, and 103 fourth-year students, as well as 118 postgraduates.

### 3.2. Design and Measures

A survey design was utilised in the present study. The survey consisted of demographic questions and the following psychological scales in Mandarin Chinese.

#### 3.2.1. Smartphone Distraction

The Chinese version of the Smartphone Distraction Scale (SDS) [9] validated by Yang et al. [10] was used to measure participants’ smartphone distraction. The scale includes 16 items, example items include “I get distracted by my phone applications”, “I get anxious if I don’t check messages immediately on my phone”, and “I use several applications on my phone while working”. The SDS consists of four subscales (attention impulsiveness, online vigilance, multitasking, and emotion regulation). Participants were required to rate on a five-point Likert scale ranging from 1 (almost never) to 5 (almost always), with higher scores indicating a higher degree of smartphone distraction. In the present study, scale reliability (Cronbach’s alpha) was 0.89. The four subscales had acceptable Cronbach’s alpha values: 0.82 (attention impulsiveness), 0.81 (online vigilance), 0.71 (multitasking), and 0.87 (emotion regulation).

#### 3.2.2. Academic Anxiety

Academic anxiety was assessed using the subscale for anxiety from the Academic Emotions Questionnaire validated in Chinese [39]. The subscale contains 7 items, example items include “I feel nervous before examination”, “I often get frustrated in my study”, and “I worry about my academic performance”. Participants were required to rate on a five-point Likert scale ranging from 1 (not at all true of me) to 5 (very true of me), with higher scores indicating higher levels of academic anxiety. Scale reliability (Cronbach’s alpha) in the present study was 0.87.

#### 3.2.3. Academic Procrastination

Academic procrastination was assessed by the first section of the Procrastination Assessment Scale-Students (PASS) [40], which was translated into Chinese and validated among Chinese students [41]. It consists of 18 items, divided into two parts: 12 items assess the levels of academic procrastination (example items include “to what degree do you procrastinate on writing a term paper?”, “to what degree is procrastination on studying for an exam a problem for you?”), and 6 items measure the intention to avoid procrastination. In this study, participants were only asked to respond to the former 12 items on a five-point Likert scale from 1 (never) to 5 (always), with higher scores indicating higher levels of academic procrastination. Scale reliability (Cronbach’s alpha) was 0.85.

#### 3.2.4. Time Management Disposition

Time management disposition was assessed by the Adolescence Time Management Disposition Inventory (ATMD) [34]. In this study, a short version of the ATMD validated in Chinese was utilized [42]. This scale includes 22 items, example items include “I usually organize my daily activities into a schedule”, “I can use my time effectively”, and “I think that time is power”. The ATMD measures three dimensions of time management disposition, including the sense of time value, the sense of time control, and the sense of time efficacy. Participants were required to rate on a five-point Likert scale ranging from 1 (not at all true of me) to 5 (very true of me), with higher scores indicating higher levels of time management disposition. Scale reliability (Cronbach’s alpha) was 0.88.

### 3.3. Procedure

Participants were recruited both online and offline. The online survey was posted on social media groups and platforms among the university students and convenience sampling was adopted when the authors distributed the paper-based survey during class breaks. Some participants introduced their classmates or friends to take the survey. At the beginning of the survey, participants were presented with a participant information page and consent page, and those consenting were then presented with the survey followed by a debrief statement. The first author’s university ethics committee approved the study. All participants were informed about the study, and all provided informed consent prior to participation.

### 3.4. Data Analysis

IBM SPSS version 26 was used for the statistical analysis. Common method biases were examined using Harman’s single-factor testing. Descriptive statistics (mean scores, standard deviations, Cronbach’s alpha) and Pearson’s correlation were calculated. Model 4 of the PROCESS macro for SPSS was used to examine the mediating effect of academic procrastination and Model 15 was applied to test the moderated mediating effect. In the present study, missing data were replaced using regression imputation.

## 4. Results

### 4.1. Preliminary Analyses

Since all of the data were from students’ self-reports, Harman’s single-factor analysis was used to examine common method biases. The results showed that the eigenvalues of 12 factors were greater than 1, and the explanatory power of the first factor was 17.08%, which was less than 40% of the critical value, indicating that the common method bias in the present study was not serious.

The descriptive statistics and correlations of the main study variables are shown in Table 1. Smartphone distraction was positively and significantly correlated with academic anxiety (*r* = 0.40, *p* < 0.001) and academic procrastination (*r* = 0.42, *p* < 0.001). Academic anxiety was positively and significantly correlated with academic procrastination (*r* = 0.39, *p* < 0.001) while time management disposition was negatively and significantly correlated with academic procrastination (*r* = −0.23, *p* < 0.001). Moreover, gender was positively and significantly correlated with smartphone distraction (*r* = 0.13, *p* < 0.01) and academic anxiety (*r* = 0.10, *p* < 0.05), indicating that females were more likely to be distracted by smartphones and had higher levels of academic anxiety than males.

### 4.2. Testing the Mediation of Academic Procrastination

To examine the effects of smartphone distraction and academic procrastination on academic anxiety, a mediation model (Model 4) with gender and age as control variables was tested. As Table 2 shows, smartphone distraction positively predicted academic anxiety (*B* = 0.23, *t* = 9.54, *p* < 0.001) and academic procrastination (*B* = 0.33, *t* = 9.84, *p* < 0.001). In the mediation model, academic anxiety was positively and significantly predicted by smartphone distraction (*B* = 0.17, *t* = 6.45, *p* < 0.001) and academic procrastination (*B* = 0.20, *t* = 6.23, *p* < 0.001). A bootstrapping analysis with 5000 bootstrap samples showed that the mediating effect of academic procrastination was significant (effect = 0.07, 95%CI [0.04, 0.10]). These results indicate that academic procrastination could partially mediate the relationship between smartphone distraction and academic anxiety. Thus, H1 and H2 were supported.

### 4.3. Testing the Moderated Mediation Model

Controlling for gender and age, Model 15 of the PROCESS macro was used to examine the moderating effect of time management disposition. As shown in Table 3, the moderated mediation model showed that smartphone distraction positively predicted academic anxiety (*B* = 0.47, *t* = 3.06, *p* < 0.01), while academic procrastination negatively predicted academic anxiety (*B* = −0.40, *t* = −2.05, *p* < 0.05). Furthermore, the interaction between smartphone distraction and time management disposition had a significant predictive effect on academic anxiety (*B* = −0.004, *t* = −2.01, *p* < 0.05), and the effect of the interaction between academic procrastination and time management disposition was also significant (*B* = 0.008, *t* = 3.19, *p* < 0.01). These results indicate that time management disposition could moderate the relationship between smartphone distraction and academic anxiety as well as the relationship between academic procrastination and academic anxiety. Thus, H3 was supported.

**Figure 2:**
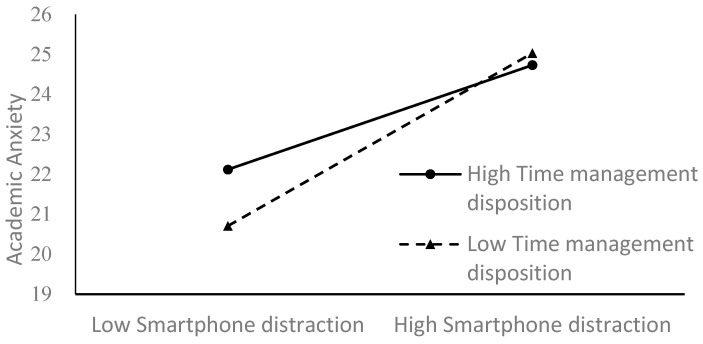
Time management disposition moderates the relationship between smartphone distraction and academic anxiety.

**Figure 3:**
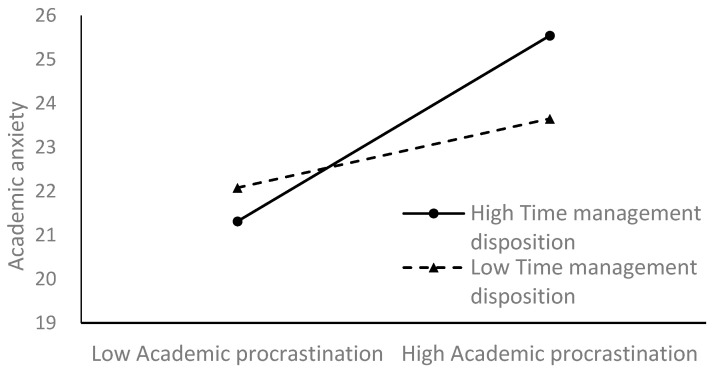
Time management disposition moderates the relationship between academic procrastination and academic anxiety.

To better illustrate the moderating effect of time management disposition, a simple slope analysis was conducted. Figure 2 shows the relationship between smartphone distraction and academic anxiety at low and high levels of time management disposition (1 SD below and above the mean). The results indicated that for students with low time management disposition, the relation was significant (*β_simple_* = 0.22, *t* = 5.97, *p* < 0.001) and for students with high time management disposition, the relation was also significant (*β_simple_* = 0.13, *t* = 4.31, *p* < 0.001) but less powerful. Figure 3 shows the relationship between academic procrastination and academic anxiety at different levels of time management disposition. There was a significant predictive association (*β_simple_* = 0.27, *t* = 7.19, *p* < 0.001) from academic procrastination to academic anxiety among students with high time management disposition and a less significant one (*β_simple_* = 0.10, *t* = 2.08, *p* < 0.05) with smaller effect size among students with low time management disposition.

The results of the conditional effect analysis are presented in Table 4 and Table 5. With the increase in the level of time management disposition, the direct effect of smartphone distraction on academic anxiety was always significant but became weak. The indirect effect of smartphone distraction on academic anxiety was significant when time management disposition was medium or high, but not when it was moderated to low. These results indicate that the mechanisms of smartphone distraction predicting academic anxiety for students with different levels of time management disposition are different.

## 5. Discussion

### 5.1. Summary of the Findings

The present study investigated the relationship between smartphone distraction, academic procrastination, academic anxiety and time management The results revealed that academic procrastination mediated the relationship between smartphone distraction and academic anxiety. Smartphone distraction predicted higher academic procrastination, which could be linked with higher academic anxiety. Moreover, time management disposition moderated the paths from smartphone distraction and academic procrastination to academic anxiety in the mediation model. For the participants with higher time management disposition, the effect of smartphone distraction on academic anxiety was smaller but the effect of academic procrastination was larger. This suggests that students with better time management skills could easily feel anxious because of their procrastination compared with those with poor time management. However, the better time managers were less affected by smartphone distraction.

### 5.2. Theoretical and Practical Implications

#### 5.2.1. The Predicting Effects of Smartphone Distraction

Previous studies suggest that smartphone distraction can predict low psychological well-being such as anxiety [10,16,20]. In line with this, the present study revealed two potential consequences of smartphone distraction, namely academic procrastination and academic anxiety, which contribute to the literature on the effects of smartphone distraction. Previous research largely proved that procrastination and anxiety are associated with PSU [4,5,6,8] but did not specify the role of smartphone distraction. The present study further explained such associations and confirmed that smartphone distraction can be the key component of PSU as discussed in previous studies [9,10]. This new and important finding from the present study proves that consideration must be given to smartphone distraction or the distracting functions of smartphones when exploring the effects and interventions of PSU. For example, the effects of distracting smartphone notifications on procrastination or anxiety might need further investigation. Interventions aimed at smartphone distraction might also need to focus on resolving the problems associated with PSU, such as decreased concentration, lower productivity or sleep problems [18].

#### 5.2.2. Academic Procrastination as a Mediator 

The mediating effect of academic procrastination between smartphone distraction and academic anxiety identified in this study contributes to the understanding of theories and empirical studies on academic procrastination. The theory of academic procrastination [23] suggests that one of the key antecedents for procrastination could be individuals’ organizational skills. The present study further explained this path; distraction caused by smartphones can be the reason for academic procrastination. This is in line with many studies that confirm PSU and procrastination are closely associated [7,11,12]. Thus, it seems possible that poor organization skills concerning specific behaviours such as smartphone use could lead to procrastination. Therefore, to understand the reasons for academic procrastination, individuals’ control over specific activities, such as smartphone use, should be considered besides general organizational skills. Furthermore, similar to previous studies [25,26,30,31], the present study identified the predictive role of procrastination on academic anxiety. This finding is in line with the theory of academic procrastination [23], which concludes that academic procrastination can predict negative emotions such as anxiety. Moreover, it is also necessary to further explore whether smartphone distraction is the result of maladaptive procrastination or the anxiety stemming from procrastination. In other words, more studies are needed on the potential “circle of smartphone distraction, procrastination, and anxiety”.

#### 5.2.3. Time Management Dispositions Moderate the Mediation Model 

The moderated mediation model revealed in this study suggests that the effects of smartphone distraction and procrastination on anxiety could be different between individuals with higher and lower time management dispositions. These findings add to the body of research on smartphone distraction, a concept that has recently emerged. The direct effects of smartphone distraction on academic anxiety are not the same for individuals with different organisational skills. This could be explained by Throuvala et al.’s findings that smartphone distraction was associated with poor self-regulation [9]. That is, individuals who cannot organise their studies or lives properly are more likely to be distracted by smartphones which could then lead to anxiety. Similar to this, previous studies found that self-control moderated the relationship between PSU and depression and anxiety [15]. Therefore, it is not always true that smartphone distraction can definitely predict anxiety or depression. Personal characteristics such as time management dispositions or self-control are potential influencing factors that should not be ignored. Furthermore, the effect of academic procrastination on academic anxiety was also moderated by time management dispositions. Interestingly, for better time managers in this study, the effect of procrastination on anxiety was larger. It is possible that the individuals with better time management (or the “good students”) are more anxious about delaying work. This could contribute to the theory of academic procrastination which suggests that delaying academic tasks could result in better working efficiency and quality of work [23]. It seems that academic procrastination can benefit working and studying because individuals or students with high time management dispositions are too worried about their unfinished tasks. 

#### 5.2.4. Procrastination and Time Management 

Previous studies found that procrastination was negatively associated with time management [36,37]. In line with this, the present study found academic procrastination was negatively correlated with time management disposition. That is, better time management means less procrastination. However, the framework of active and passive procrastination [43,44] suggests that procrastination does not always indicate poor learning strategy. Apart from the negative perspective of procrastination, active procrastination was defined as the intentional delaying behaviours of individuals who can deal with time pressure well and meet the deadlines successfully [44]. Therefore, it is necessary to further explore whether better time management predicts lower academic procrastination in general or lower passive procrastination.

### 5.3. Limitations and Future Directions

There are several limitations of the present study. (1) Only self-reported data were collected in this study; socially desirable answers are inevitable. Causal relationships could not be established and studies in the future could adopt longitudinal designs. (2) Furthermore, theoretical evidence suggests that anxiety can be affected by environmental factors or the context of learning [21,22]. The present study might have ignored contextual factors such as exams or deadlines during the data collection process. Future studies could attempt to use experimental designs to explore the cognitive and neural mechanisms of smartphone distraction. (3) The non-homogeneity of the sample might be a limitation. Future studies should focus on recruiting participants from specific age groups or consider a sample with a wider range of age categories. Cultural variability might be an essential factor to take into account. (4) The study did not delve into the effects of various technological settings, such as persistent notifications, or environmental factors, such as the presence of Wi-Fi. Further studies could focus on the impacts of such environmental factors on smartphone distraction. (5) In addition, the different types of academic activities undertaken by participants (e.g., reading versus practical tasks) could uniquely impact smartphone distraction and anxiety. Another future direction is to examine how smartphone distractions affect various kinds of academic tasks and related emotions. 

## 6. Conclusions

The present study’s findings provided unique insights into smartphone distraction and its associations with academic procrastination and academic anxiety. The findings also revealed the important role of different time management dispositions. The findings will be of benefit to students and employers. The findings may inform the development of prevention and treatment programmes that focus on anxiety. Support programmes could concentrate on fostering self-regulation and time management skills, advancing digital literacy, and promoting a mindful approach to technology use. Establishing supportive study environments with fewer distractions (e.g., using smartphones silently in study areas) is also crucial for students’ academic emotions and achievements.

## Figures and Tables

**Table 1:** Descriptive statistics and correlations for the main study variables (N = 474).

	*Mean*	*SD*	1	2	3	4	5	6
1. Gender	1.85	0.36	-					
2. Age	21.25	2.04	0.01	-				
3. Smartphone distraction	53.61	9.98	0.13 **	0.10 *	-			
4. Academic anxiety	23.02	5.71	0.10 *	−0.07	0.40 ***	-		
5. Academic procrastination	36.52	7.83	0.06	0.06	0.42 ***	0.39 ***	-	
6. Time management disposition	77.05	11.16	0.00	0.07	−0.07	−0.03	−0.23 ***	-

Note. * *p* < 0.05, ** *p* < 0.01, *** *p* < 0.001 Gender (1 = male, 2 = female).

**Table 2:** Testing the mediation model (N = 474).

Predictors	Model 1 (Academic Anxiety)		Model 2 (Academic Procrastination)		Model 3 (Academic Anxiety)	
	*B*	*t*	*B*	*t*	*B*	*t*
Gender	0.75	1.11	0.05	0.06	0.73	1.14
Age	−0.30	−2.57	0.06	0.36	−0.32	−2.77 **
Smartphone distraction	0.23	9.54 ***	0.33	9.84 ***	0.17	6.45 ***
Academic procrastination					0.20	6.23 ***
*R* ^2^	0.17		0.18		0.24	
*F*	33.08 ***		33.53 ***		36.49 ***	

Note. ** *p* < 0.01, *** *p* < 0.001.

**Table 3:** Testing the moderated mediation model (N = 474).

Predictors	*R* ^2^	*F*	*B*	*t*
Model (academic anxiety)	0.26	23.02 ***		
Gender			0.66	1.03
Age			−0.31	−2.77 **
Smartphone distraction			0.47	3.06 **
Academic procrastination			−0.40	−2.05 *
Time management disposition			−0.05	−0.48
Smartphone distraction × Time management disposition			−0.004	−2.01 *
Academic procrastination × Time management disposition			0.008	3.19 **

Note. * *p* < 0.05, ** *p* < 0.01, *** *p* < 0.001.

**Table 4:** Conditional direct effect of smartphone distraction on academic anxiety.

Time Management Disposition	Effect	SE	LLCI	ULCI
M − 1SD (65.89)	0.22	0.04	0.15	0.29
M (77.05)	0.17	0.03	0.12	0.22
M + 1SD (88.21)	0.13	0.03	0.07	0.19

Note. M = mean, SD = Standard deviation, SE = Standard error, LLCI = Lower Limit of Confidence Interval, ULCI = Upper Limit of Confidence Interval.

**Table 5:** Conditional indirect effect of smartphone distraction on academic anxiety.

Time Management Disposition	Effect	BootSE	BootLLCI	BootULCI
M − 1SD (65.89)	0.03	0.02	−0.01	0.07
M (77.05)	0.06	0.01	0.03	0.09
M + 1SD (88.21)	0.09	0.02	0.05	0.13

Note. Bootstrap sample size = 5000, SE = Standard error, LLCI = Lower Limit of Confidence Interval, ULCI = Upper Limit of Confidence Interval.

## Data Availability

The data are not publicly available due to privacy.
